# P158: Posting a management dashboard improves behaviour in the OR

**DOI:** 10.1186/2047-2994-2-S1-P158

**Published:** 2013-06-20

**Authors:** R Meinke, OT Reuthebuch, J Fassl, I Gisler, M Heiberger, M Seeberger, FS Eckstein, AF Widmer

**Affiliations:** 1 Infectious Diseases and Hospital Epidemiology, University Hospital Basel, Basel, Switzerland; 2 Cardiac Surgery, University Hospital Basel, Basel, Switzerland; 3 Anaesthesiology, University Hospital Basel, Basel, Switzerland

## Introduction

Surgical site infections (SSIs) lead to increased morbidity and mortality. Guidelines to prevent SSIs have been issued, but adherence is commonly low. Interventions in the OR to improve adherence are frequently short term. A dashboard is a management tool used in industries showing defined performance indicators at a glance, it is easily accessible to everyone and recipients are able to influence the indicators. We tested this approach in cardiac surgery with defined process parameters that were regularly monitored.

## Methods

In interdisciplinary team-meetings, all process indicators were discussed, and a well-defined set approved, all being recommended by WHO. The dashboard was posted monthly at the OR entry, and reported to the cardiac-thoracic-surgery-team in 2-monthly meetings. The survey period lasted from 10/2011-11/2012.

### Parameters and aims

- Timing of Antibiotic-prophylaxis (30-60 min before incision)

- Preoperative temperature (core temperature > 36°C prior incision)

- Discipline in the OR (hand-disinfection performed at 5 moments WHO; no jewellery/or covered; correct wearing of surgical mask)

The indicators were surveyed by ~4 control-visits in the OR per month as well as by analysis of data from the electronic OR chart. The feedback of adherence was simplified by using a traffic-light-system that was implemented to show parameters at a glance and posted monthly at the OR-doors:

### Colour-system

- RED (not fulfilled; Score 0 points)

- ORANGE (limited adherence; score 1 point)

- GREEN (fulfilled; score 2 points)

Maximal achievable points are 16.

## Results

Analysis of parameters showed an increase in compliance over time, from an average of 7 points at start of the survey-period to an average of 15 (p<0.05). However, two time periods showed decreased adherence, but rapidly exceeded the level of previous months.

## Conclusion

The very simple dashboard provided rapid and easy feedback on compliance to guidelines. It was readily accepted by members of the interdisciplinary team, and helped to improve the teams’ performance. If supported by senior staff and open discussion of not fulfilled parameters this tool helps to sustain high levels of adherence after an initial intervention.

## Disclosure of interest

None declared

